# The triglyceride glucose-waist-to-height ratio outperforms obesity and other triglyceride-related parameters in detecting prediabetes in normal-weight Qatari adults: A cross-sectional study

**DOI:** 10.3389/fpubh.2023.1086771

**Published:** 2023-04-06

**Authors:** Neyla S. Al Akl, Elias N. Haoudi, Halima Bensmail, Abdelilah Arredouani

**Affiliations:** ^1^Qatar Foundation, Diabetes Research Center, Qatar Biomedical Research Institute (QBRI), Hamad Bin Khalifa University (HBKU), Doha, Qatar; ^2^Qatar Foundation, Qatar Academy Doha, Doha, Qatar; ^3^Qatar Computing Research Institute, Hamad Bin Khalifa University, Doha, Qatar; ^4^Qatar Foundation, College of Health and Life Sciences, Hamad Bin Khalifa University (HBKU), Doha, Qatar

**Keywords:** prediabetes, diabetes, triglyceride-glucose-related waist-to-height ratio, obesity, normal-weight, Qatar

## Abstract

**Introduction:**

The triglyceride-glucose (TyG)-driven indices, incorporating obesity indices, have been proposed as reliable markers of insulin resistance and related comorbidities such as diabetes. This study evaluated the effectiveness of these indices in detecting prediabetes in normal-weight individuals from a Middle Eastern population.

**Methods:**

Using the data of 5,996 adult Qatari participants from the Qatar Biobank cohort, we employed adjusted logistic regression to assess the ability of various obesity and triglyceride-related indices to detect prediabetes in normal-weight (18.5 ≤ BMI <25 kg/m^2^) adults (≥18 years).

**Results:**

Of the normal-weight adults, 13.62% had prediabetes. TyG-waist-to-height ratio (TyG-WHTR) was significantly associated with prediabetes among normal-weight men [OR per 1-SD 2.68; 95% CI (1.67–4.32)] and women [OR per 1-SD 2.82; 95% CI (1.61–4.94)]. Compared with other indices, TyG-WHTR had the highest area under the curve (AUC) value for prediabetes in men [AUC: 0.76, 95% CI (0.70–0.81)] and women [AUC: 0.73, 95% CI (0.66–0.80)], and performed significantly higher than other indices (*p* < 0.05) in detecting prediabetes in men. Tyg-WHTR shared similar diagnostic values as fasting plasma glucose (FPG).

**Discussion:**

Our findings suggest that the TyG-WHTR index could be a better indicator of prediabetes for general clinical usage in normal weight Qatari adult men than other obesity and TyG-related indices. TyG-WHTR can help identify a person’s risk for developing prediabetes in both men and women when combined with FPG results.

## Introduction

Although most normal-weight adults with BMI between 18.5 and 25 kg/m^2^ are seemingly healthy, a significant number of them may be affected by undiagnosed metabolic disorders such as insulin resistance, prediabetes, type 2 diabetes (T2D), and nonalcoholic fatty liver disease (NAFLD) (1). These individuals are classified as Normal-Weight Obese (NWO) because they usually have a high body fat mass but a normal BMI (2). Because of their increased risk of cardiometabolic morbidity and mortality, there is a growing interest in this group of subjects (2–4). The global prevalence of NWO ranges from 4.5 to 22% due to the wide variation in body fat percent cut-offs used to diagnose excess body fat in different populations (5). The exact etiology of NWO is unclear, but genetics, diet, and physical activity have all been associated with the condition. Compared to normal-weight lean (NWL) subjects with a normal BMI and body fat amount, the NOW subjects present changes in body composition, inflammation, and oxidative stress (2).

Screening for prediabetes and diabetes is recommended in overweight or obese adults (6). However, given the prevalence of NWO, these disorders may go undiagnosed in individuals with a seemingly normal weight. Prediabetes is a subclinical high-risk state that could lead to diabetes and conventional diabetes complications (7). Prediabetes is associated with the concomitant presence of insulin resistance and β-cell dysfunction, instigated before detectable glucose modifications (8). Prediabetes is defined as having a Hb1A_c_ level between 5.7 and 6.4% (39 and 47 mmol/mol), a fasting glucose concentration between 100 and 125 mg/dL (5.6 and 6.9 mmol/L), or a 2 h oral glucose tolerance test between 140 and 200 mg/dL (7.8–11.0 mmol/L) (6). Prediabetes affected 7.5% of the global population in 2019, and this figure is expected to rise to 8.6% by 2045 if no prompt actions are taken (Saeedi, 2019 #5).

According to previous prospective studies, the annualized conversion rate from prediabetes to diabetes is between 5 and 10% (8, 9). Additionally, persons with prediabetes have a 6-year risk of T2D at a rate of 33–65%, compared to 5% of those with normoglycemia (10).

Reports from the Middle East region show that, like T2D, prediabetes is highly prevalent in the region’s nations, with rates ranging from 20 to 40% (11, 12). Fortunately, many people with prediabetes can revert to normoglycemia and prevent the progression to T2D in response to sustained lifestyle changes and/or medication (13–16). Hence, identifying convenient clinical markers that can efficiently diagnose prediabetes would benefit from closer monitoring and early intervention to prevent T2D onset.

To date, the gold standard test for prediabetes diagnosis is the oral glucose tolerance test (OGTT), fasting plasma glucose (FPG), and HbA_1c_ levels (11, 12). However, several studies have shown discordance between HbA_1c_ and glycemia (17–21). This discordance could have a significant impact on clinical practice. Consequently, there is a need for improved and more reliable diagnostic tools for prediabetes and diabetes. Numerous obesity indices (22)such as waist circumference (WC) (23), waist-to-height ratio (WHR) (24), Visceral Adiposity Index (VAI) (12) and lipid accumulation product (LAP) (12, 22) have had their potential in predicting diabetes. However, many promising surrogate indices are being studied for predicting diabetes and performed better than the traditional markers identified hereinabove. These markers include the triglyceride glucose (TyG)-related parameters (TyG), TyG–Body mass index (TyG-BMI), TyG-WC, and TyG-WHTR (25–30). Some epidemiological studies also targeted a few surrogate indices in predicting prediabetes. Wen et al. found that TyG performed better as an indicator for prediabetes than the conventional markers in the Chinese Elderly (31). TyG also scored higher as a predictive index for prediabetes among reproductive-age women (32). Furthermore, the TyG index was useful as a surrogate tool to estimate dysglycemia in obese adolescents (33, 34).

To the best of our knowledge, no previous study has investigated the association of triglyceride glucose (TyG)-related indices with prediabetes in a population from the Middle East. Consequently, this cross-sectional study sought to investigate the association of TyG-glucose-related parameters with prediabetes, especially in normal-weight individuals, and evaluate their superiority over conventional dysglycemia risk factors.

## Methods

### Study population

We obtained cross-sectional clinical, anthropometric, and demographic data of 5,996 Qatari individuals aged between 18 and 86 years and collected between 2012 and 2020 by the Qatar Biobank (QBB), a national institute running a well-phenotyped cohort of individuals from the general population (35). Based on the American Diabetes Association guidelines, 1996 (33.2%) of the 5,996 subjects had prediabetes, defined as having HbA1c levels between 39 mmol/mol (5.7%) and 47 mmol/mol (6.4%).

### Anthropometric and clinical measures

Plasma samples of patients fasting for at least 6 h were handled according to a standard protocol within 2 h of blood collection. FPG, HbA1c, triglyceride (TG), total cholesterol (TC), low-density lipid cholesterol (LDL-C), and high-density lipid cholesterol (HDL-C) were analyzed with an automated biochemical analyzer at the central laboratories at the Hamad Medical Corporation in Doha. Bodyweight (kg) and height (cm) were measured in a standing position. BMI (kg/m^2^) was calculated as weight (kg) divided by the square of height (m). We used Caucasian BMI cut-off values to categorize BMI into two groups: normal-weight (BMI 18.5–24.9 kg/m^2^) and overweight/obese (BMI ≥25 kg/m^2^). Prediabetes cases were defined as those individuals with HbA1_c_ between 39 mmol/mol (5.7%) and 47 mmol/mol (6.4%), whereas controls were those with HbA1_c_ < 39 mmol/mol (5.7%). An informed written consent to use collected data for research was obtained by the QBB for all the participants. The present project was approved by the IRB of the Qatar biobank (protocol Ex-2018-Res-ACC-0123-0067).

### Definitions of obesity and triglyceride indices

We used the formulas in the table below to define the obesity or TyG-related indices.

### Statistical analysis

The subjects were divided into groups for statistical analysis based on gender, prediabetes presence or absence, BMI, and age as needed.

Data analyses were performed using Stata/IC 16.1 software.^1^ Descriptive statistics were used to compare the baseline characteristics of the participants. Variables with outliers were winsorized using winsor2 command in Stata. Continuous variables were expressed as means ± standard deviation (SD) and compared using the independent sample T-test between the two groups. Categorical variables were expressed as percentages, and the Chi-squared test was employed to compare two groups. The odds of prediabetes were determined by binary logistic regression, using the continuous variables for both obesity and triglyceride indices as independent variables. Odd ratios (ORs) were standardized by using transformed observations [(observation − mean)/SD] in the models. Results are presented as Odds Ratios (OR) with associated 95% confidence intervals (CI) for 1-SD increase of the independent variables. The predictive value for prediabetes of each index was determined by the area under the curve (AUC) in the Receiver operating characteristic curve (ROC) analyses. The cut-off point was selected according to the Youden index (sensitivity + specificity −1). Statistical significance was set at *p* < 0.05.

## Results

### Demographic and clinical characteristics of participants

Table 1 displays the baseline characteristics of the participants. Of the 5,996 individuals, 1996 had prediabetes (HbA_1c_ between 5.7 and 6.5%). The mean age of normoglycemic subjects and those with prediabetes was 36.37 and 48.17 years old, respectively (*p* < 0.001). Women represented 53.9% of the normoglycemic subjects and 53.3% of the subjects with prediabetes. Subjects with prediabetes showed significantly higher obesity and TyG-related indices than those with normoglycemia (*p* < 0.05).

**Table 1:** Obesity and TyG-related indices.

Obesity indices			
Index name	Abbreviation	Formula	References
Waist-to-height ratio	WHTR	WHTR = Waist _(cm)_/Height _(cm)_	(36)
Visceral Adiposity Index	VAI (Men)	VAI_(men)_ = [(WC _(in cm)_/39.68) + (1.88 × BMI _(in Kg/m2)_)] × [(TG _(in mmol/L)_/1.03) × (1.31/HDL-C _(in mmol/L)_)]	(37)
Visceral Adiposity Index	VAI (Women)	VAI_(women)_ = [(WC _(in cm)_ /36.58) + (1.89 × BMI _(in Kg/m2)_)] × [(TG _(in mmol/L)_/0.81) × (1.52/HDL-C _(in mmol/L)_)]	(37)
Lipid accumulation product	LAP (Men)	LAP _(men)_ = [WC _(in cm)_-65] × TG _(in mmol/L)_	(38)
Lipid accumulation product	LAP (Women)	LAP _(women)_ = [WC _(in cm)_-58] × TG _(in mmol/L)_	(38)
TyG related parameters			
Triglyceride-glucose	TyG	TyG = Ln [(TG _(in mg/dL)_ × FBG _(in mg/dL)_/2)]	(39)
Triglyceride-glucose-BMI	TyG-BMI	TyG-BMI = TyG × BMI	(27)
Triglyceride-glucose-waist circumference	TyG-WC	TyG-WC = TyG × WC	(27)
Triglyceride-glucose-waist-to-height ratio	TyG-WHTR	TyG-WHTR = TyG × WHTR	(27)

### Associations of indicators with prediabetes risk

Gender-specific multivariate logistic regression models were fitted for each indicator variable to calculate the age-adjusted OR (aOR) per 1-SD with 95% CI for prediabetes. The aORs per 1-SD for the obesity and TyG indices were significant in both men and women (Table 2). Among the tested indices, TyG-WHTR and TyG-WC showed the strongest association with prediabetes [TyG-WHTR: aOR 2.19; 95%CI (1.96–2.46) in men and aOR 2.76; 95%CI (2.30–2.86) in women; TyG-WC: aOR 2.08; 95% (1.87–2.32) in men and aOR 2.68; 95% (2.38–3.02) in women].

**Table 2:** Baseline demographic and clinical characteristics of the participants.

	NG (*n* = 4,000)	Prediabetes (*n* = 1996)	*p*-value
Men/Women	1842/2158	928/1068	
Age	36.37 ± 10.42	48.17 ± 11.39	<0.0001
BMI (Kg/m^2^)	28.45 ± 5.58	32.02 ± 5.58	<0.0001
NW/(Ow + Ob)	1090/2910	172/1824	<0.0001
SBP (mm Hg)	111.11 ± 12.93	121.67 ± 15.00	<0.0001
DBP (mm Hg)	66.94 ± 9.89	72.22 ± 10.55	<0.0001
TC (mmol/L)	4.94 ± 0.89	5.15 ± 0.94	<0.0001
TG (mmol/L)	1.17 ± 0.64	1.49 ± 0.72	<0.0001
HDL-C (mmol/L)	1.41 ± 0.37	1.31 ± 0.34	<0.0001
LDL-C (mmol/L)	2.99 ± 0.82	3.16 ± 0.88	<0.0001
FPG (mmol/L)	4.88 ± 0.56	5.60 ± 0.89	<0.0001
HbA_1c_ (%)	5.17 ± 0.3	5.9 ± 0.21	<0.0001
**TyG-related parameters**			
TyG	8.29 ± 0.51	8.68 ± 0.49	<0.0001
TyG-BMI	236.7 ± 51.79	278.35 ± 51.26	<0.0001
TyG- WC	714.85 ± 131.48	834.09 ± 122.77	<0.0001
TyG-WHTR	4.33 ± 0.75	5.11 ± 0.72	<0.0001
**Obesity indices**			
WC (cm)	85.86 ± 12.92	95 ± 12.11	<0.0001
WHTR	0.52 ± 0.07	0.58 ± 0.07	<0.0001
VAI	1.38 ± 1.08	1.94 ± 1.30	<0.0001
LAP	31.17 ± 26.88	52.46 ± 32.22	<0.0001

Values are presented as range, mean ± SD, or frequencies (%). NW, normal-weight; Ow + Ob, overweight + obese; NG, Normoglycemic; BMI, body mass index; FPG, fasting plasma glucose; HDL-C, high-density lipoproteins; LDL-C, low-density lipoproteins; TC, total cholesterol; TG, total triglycerides; SBP, Systolic blood pressure; DBP, Diastolic blood pressure; WC, Waist Circumference; WHTR Waist Height-Ratio; VAI, Visceral Adiposity Index; LAP Lipid Accumulation Product; TyG, Triglyceride Glucose; TyG-BMI, TyG related to BMI; TyG-WC TyG related to WC; TyG-WHTR, TyG related to WHTR. Statistical significance is considered at *p* < 0.05.

**Figure 1:**
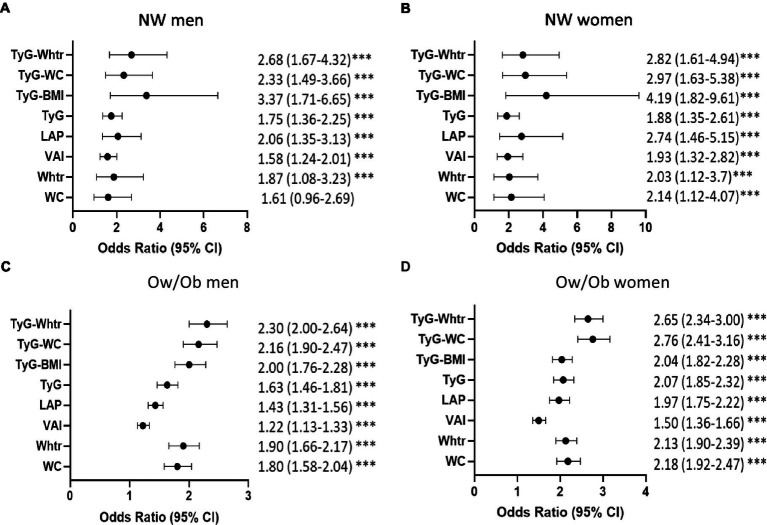
Strength of association of obesity and triglyceride indices with prediabetes in normal-weight and overweight/obese individuals. Age-adjusted Odds Ratios and 95%CI for prediabetes in each index by per 1-SD in NW men **(A)**, NW women **(B)**, Ow/Ob men **(C)**, Ow/Ob women **(D)**. ***p < 0.001. Statistical significance is considered at *p* < 0.05. *** indicates *p* < 0.001.

Further stratification of subjects by BMI resulted in higher aORs per 1-SD for prediabetes in the normal-weight (NW) individuals compared to overweight/obese (Ow/Ob) for most indices, mainly Tyg-BMI, TyG-WC, and TyG-WHTR (Figure 1). In NW men and women, TyG-BMI had the highest aOR for prediabetes [aOR 3.37; 95%CI (1.71–6.65) and aOR 4.19; 95%CI (1.82–9.61) in NW men and NW women, respectively]. TyG-WHTR and TyG-WC indices were also strongly associated with prediabetes [TyG-WHTR: aOR, 2.68; 95% CI (1.67–4.32) in NW men and aOR, 2.82; 95%CI (1.61–4.94) in NW women; TyG-WC: aOR, 2.33; 95% CI (1.49–3.66) in NW men and aOR, 2.97; 95% (1.63–5.38) in NW women]. In Ow/Ob individuals, TyG-WC and TyG-WHTR had the highest aORs in men and women compared to all other indices [TyG-WC: aOR 2.16; 95% CI (1.90–2.47) in Ow/Ob men and aOR, 2.76; 95%CI (2.41–3.16) in Ow/Ob women; TyG-WHTR: aOR, 2.30; 95%CI (2.00–2.64) in Ow/Ob men and aOR, 2.65; 95% CI (2.34–3.00) in Ow/Ob women].

### The predictive value of each index for prediabetes in normal-weight individuals

**Figure 2:**
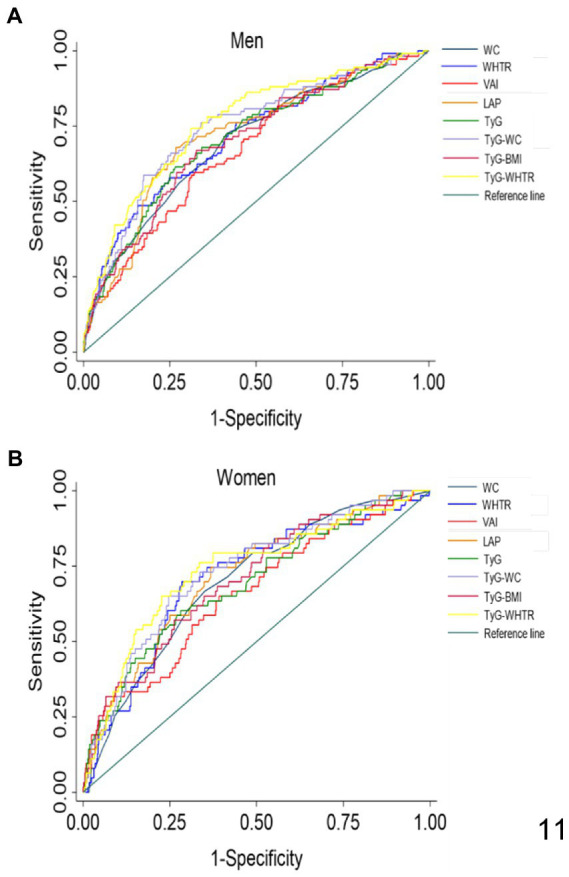
ROC curves for each index as predictors of prediabetes in normal-weight men **(A)** and women **(B)**. WC, Waist Circumference; WHTR, Waist Height-Ratio; VAI, Visceral Adiposity Index; LAP, lipid accumulation product; TyG, Triglyceride Glucose; TyG-BMI, TyG related to BMI; TyG-WC, TyG related to WC; TyG-WHTR, TyG related to WHTR.

We performed ROC analysis to assess the predictive value of each index for prediabetes in NW individuals. The results of the ROC curve analysis for each index are shown in Table 3 and Figure 2. The largest AUC observed in NW men corresponded to TyG-WHTR index [AUC: 0.76, 95% CI (0.70–0.81)] followed by TyG-WC [AUC: 0.74, 95% CI (0.69–0.79)]. The indices with the highest predictive value for prediabetes in NW women were TyG-WHTR [AUC: 0.73, 95% CI (0.66–0.80)] and TyG-WC [AUC: 0.73, 95% CI (0.66–0.79)]. TyG BMI and TyG showed approximately similar predictive ability when predicting prediabetes in normal-weight men and women [AUC ranging between (0.69 and 0.70)]. When predicting prediabetes, TyG-WC and TyG-WHTR had the highest sensitivity (76% for TyG-WC and 74% for TyG-WHTR) and Youden index (0.421 for TyG-WC and 0.432 for TyG-WHTR) in NW men. In NW women, LAP and TyG-WHTR had the highest sensitivity (75% for LAP and 76% for TyG-WHTR), and the WHTR and TyG-WHTR had the highest Youden index (0.413 for WHTR and 0.427 for TyG-WHTR).

**Table 3:** ROC curve analyses for each index in predicting prediabetes in NW participants stratified by gender.

	AUC (95%CI)	*p*-value	Cut-off	Sens (%)	Spec (%)	Youden index
**Men (620)**						
WC	0.69 (0.64–0.75)	<0.0001	≥79.5	72%	59%	0.310
WHTR	0.71 (0.65–0.76)	<0.0001	≥0.47	58%	75%	0.329
VAI	0.66 (0.61–0.72)	<0.0001	≥1.15	60%	64%	0.275
LAP	0.71 (0.66–0.77)	<0.0001	≥18.8	68%	73%	0.411
TyG	0.70 (0.65–0.76)	<0.0001	≥ 8.49	61%	73%	0.349
TyG-BMI	0.69 (0.64–0.75)	<0.0001	≥194	67%	67%	0.343
TyG-WC	0.74 (0.69–0.79)	<0.0001	≥669	76%	66%	0.421
TyG-WHTR	0.76 (0.70–0.81)	<0.0001	≥3.92	74%	69%	0.432
**Women (642)**						
WC	0.69 (0.63–0.76)	<0.0001	≥71.5	67%	65%	0.316
WHTR	0.70 (0.63–0.77)	<0.0001	≥0.45	70%	72%	0.413
VAI	0.65 (0.58–0.73)	<0.0001	≥0.87	65%	61%	0.259
LAP	0.71 (0.64–0.78)	<0.0001	≥10.57	75%	63%	0.378
TyG	0.69 (0.61–0.76)	<0.0001	≥8.19	60%	72%	0.322
TyG-BMI	0.70 (0.63–0.77)	<0.0001	≥191	57%	74%	0.309
TyG-WC	0.73 (0.66–0.79)	<0.0001	≥593	65%	75%	0.404
TyG-WHTR	0.73 (0.66–0.80)	<0.0001	≥3.62	76%	66%	0.427

WC, waist circumference; WHTR, waist height-ratio; VAI, Visceral Adiposity Index; LAP, lipid accumulation product; TyG, triglyceride glucose; TyG-BMI, TyG related to BMI; TyG-WC, TyG related to WC; TyG-WHTR, TyG related to WHTR. Statistical significance is considered at *p* < 0.05.

### Tyg-WHTR index had the highest ability to predict prediabetes

The index with the highest AUC value was contrasted with the other indices in the ROC analysis to determine the superior indicator for prediabetes (Table 4). The AUC of the TyG-WHTR index for prediabetes was significantly higher than all other indices in NW men (*p* < 0.05). In women, the predictive value of TyG-WHTR was significantly higher than the AUC of WC (*p* < 0.004), WHTR (*p* < 0.049), VAI (*p* < 0.008). However, TyG-WHTR was not significantly different from the other TyG-related indices and LAP (*p* > 0.05).

**Table 4:** Pairwise comparison of AUC of TyG-WHTR in NW participants.

	Men (620)			Women (642)		
	Differences between AUC	95% CI	*p*-value	Differences between AUCs	95% CI	*p*-value
WC	0.08	(0.04–0.11)	<0.0001	0.06	(0.01–0.10)	0.004
WHTR	0.04	(0.015–0.07)	0.0029	0.02	(0.0007–0.058)	0.049
VAI	0.09	(0.05–0.13)	<0.0001	0.07	0.02–0.13	0.008
LAP	0.04	(0.02–0.06)	<0.0001	0.02	(−0.004–0.04)	0.1
TyG	0.05	(0.01–0.09)	0.0031	0.04	(−0.005–0.09)	0.78
TyG-BMI	0.06	(0.03–0.09)	<0.0001	0.03	(−0.012–0.07)	0.15
TyG-WC	0.016	(0.0003–0.32)	0.045	0.003	(−0.016–0.024)	0.72

NW, normal-weight; AUC, area under the curve; WC, waist circumference; WHTR, waist height-ratio; VAI, Visceral Adiposity Index; LAP, lipid accumulation product; TyG, triglyceride glucose; TyG-BMI, TyG related to BMI; TyG-WC, TyG related to WC; TyG-WHTR, TyG related to WHTR. Statistical significance is considered at *p* < 0.05.

Since FPG levels are one of the determinants for prediabetes diagnosis, subgroup analyses were conducted to assess whether Tyg-WHTR and FPG indices might differentially predict prediabetes. The OR and the AUC of TyG -WHTR for prediabetes were compared to that of FPG in different subgroups, as shown in Table 5. TyG WHTR performed similarly to FPG in NW men [TyG-WHTR: AUC 0.76, 95% CI (0.70–0.81) versus FPG: AUC 0.76, 95% CI (0.72–0.81), *p* = 0.76] and women [TyG-WHTR AUC 0.73, 95% CI (0.66–0.80) versus FPG AUC 0.68, 95% CI (0.61–0.76), *p* = 0.13]. Similarly, no significant difference was found in Ow/Ob men [TyG-WHTR: AUC 0.71, 95% CI (0.69–0.74) versus FPG: AUC 0.74, 95% (0.72–0.76), *p* = 0.4]. In contrast, the predictive value of the TyG-WHTR index was significantly higher than FPG in obese women [TyG-WHTR: AUC 0.77, 95% CI (0.75–0.78) versus FPG: AUC 0.75, 95% CI (0.73–0.77) *p* = 0.0034].

**Table 5:** Performance of the TyG-WHTR index versus FPG using adjusted logistic regression and ROC analysis in predicting prediabetes in subgroups with the different characteristics.

	OR (95%)	AUC (95%CI)	Cut-off point	*p*-value
**NW men (*n*** **= 620)**				
Tyg-WHTR	2.68 (1.67–4.32)***	0.76 (0.70–0.81)	3.92	Ref
FPG	2.32 (1.73–3.10)***	0.76 (0.72–0.81)	5.11	0.76
**NW women (*n*** **= 642)**				
Tyg-WHTR	2.82 (1.61–4.94)***	0.73 (0.66–0.80)	3.62	Ref
FPG	1.90 (1.32–2.72)***	0.68 (0.61–0.76)	4.89	0.13
**Ow/Ob men (*n*** **= 2,150)**				
Tyg-WHTR	2.30 (2.00–2.64)***	0.71 (0.69–0.74)	4.94	Ref
FPG	2.35 (2.08–2.66)***	0.74 (0.72–0.76)	5.33	0.4
**Ow/Ob women (*n*** **= 2,584)**				
Tyg-WHTR	2.65 (2.34–3.00)***	0.77 (0.75–0.78)	4.73	Ref
FPG	2.61 (2.31–2.95)***	0.75 (0.73–0.77)	5.29	**0.0034**

NW, normal-weight; Ow/Ob, overweight/obese; OR, odds ratio; AUC, area under the curve; TyG-WHTR, TyG related to waist height-ratio; FPG, fasting plasma glucose. Statistical significance is considered at *p* < 0.05. ***p < 0.001.

**Figure 3:**
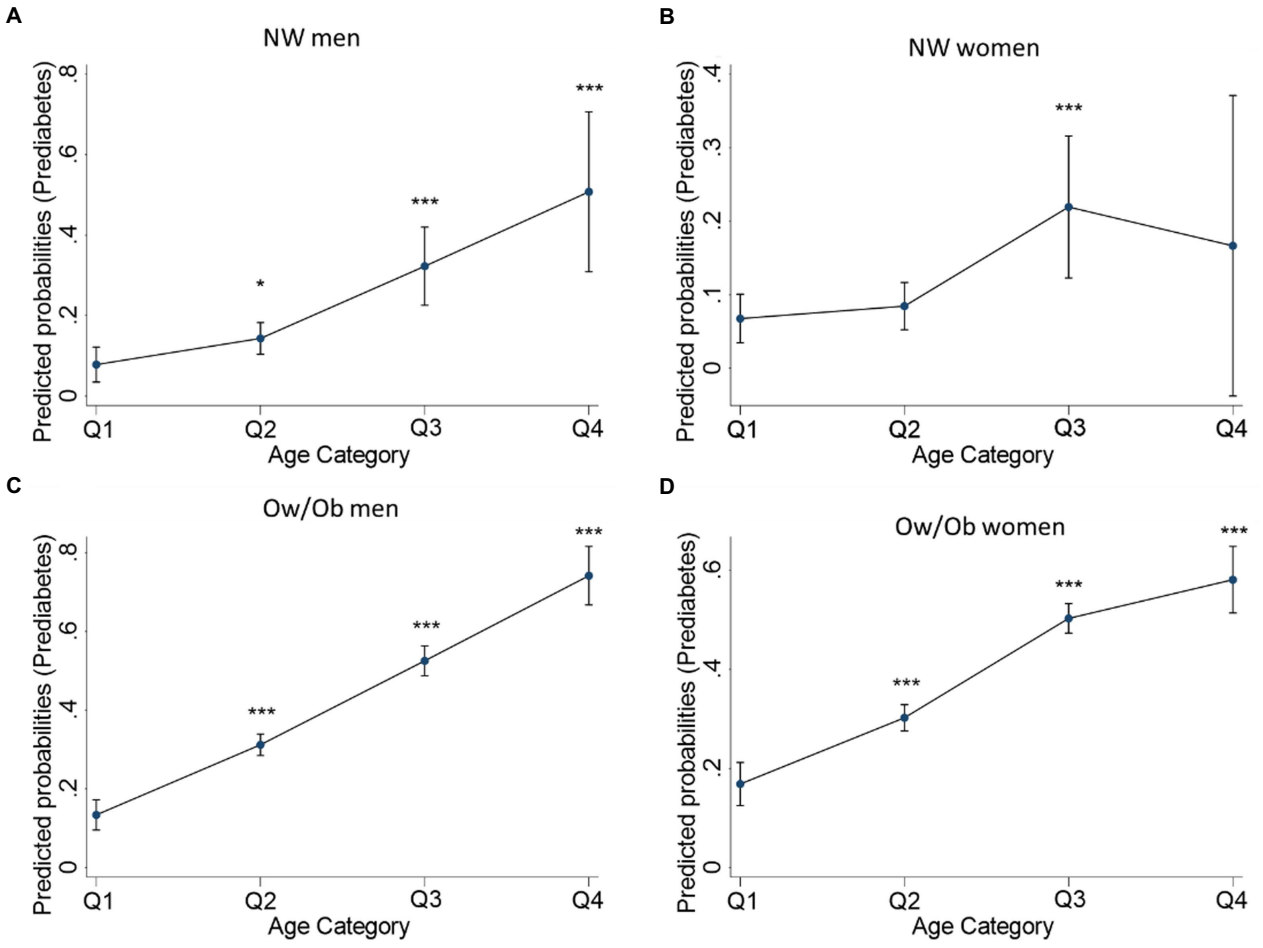
TyG-WHTR predicted probabilities for having prediabetes across age quartiles in NW and Ow/Ob participants. Predicted probabilities for Prediabetes in NW men **(A)**, NW women **(B)** Ow/Ob men **(C)**, Ow/Ob women **(D)**. Statistical significance is considered at *p* < 0.05. *p < 0.05, **p < 0.01 ***p<0.001.

We then estimated the prediabetes risk probabilities of TyG-WHTR based on four age subgroups: less than 30 years old (Q1), between 30 and less than 45 years old (Q2), between 45 and less than 60 years old (Q3), and > 60 years (Q4) (Figure 3). The results indicate that the probability of having prediabetes increases gradually and significantly with age in NW men and OW/Ob men and women but not in NW women.

## Discussion

The current study aimed to compare how well obesity and TyG-related indices might detect prediabetes in Qatari people who were normal-weight (NW). In NW men, TyG-WHTR had the highest predictive value compared to all other and most obesity indices in women. Furthermore, TyG-WHTR performed similarly to the conventionally adapted FPG index in normal-weight men and women, as well as in obese men, but was superior to FPG in obese women. These results suggest using the TyG-WHTR index as a potential predictor of prediabetes for general clinical usage in normal-weight men. When coupled with FPG results, TyG-WHTR can further determine prediabetes predisposition in both men and women. In addition, our results enabled us to determine optimal cut-off points of WC and WHTR for identifying prediabetes in normal-weight Qatari adults; WC ≥ 79.5 cm and WHTR ≥ 0.47 for men, and WC ≥ 71.5 cm and WHTR ≥ 0.45 for women.

According to the World Health Organization, the WC cut-off point for various metabolic disorders varies by ethnicity (40). Although, the present data facilitated the identification of the WC and WHTR cut-off points needed for predicting prediabetes risk in a Middle Eastern population, a person’s height can affect the predictive ability of WC (36). As a result, adopting the WHTR marker outweighed BMI and WC in predicting certain metabolic diseases, including diabetes (26, 41, 42). In line with these findings, our results revealed that the association of WHTR with prediabetes was stronger than WC among the normal-weight adult population.

Furthermore, WHTR had higher specificity in normal-weight men and women than WC and higher sensitivity in women. This result suggests that WHTR may have a better predictive ability than WC and may be used to screen for prediabetes. Although these anthropometric parameters performed poorly than the TyG-WHTR index, as indicated by the ROC curve analyses, their predictive value was equivalent to LAP, VAI, and the other TyG-related indices. These findings point to the inclusion of these predictors collectively to improve the accuracy of prediabetes diagnosis over anthropometric indices alone.

Obesity increases the risk of numerous chronic disease, such as T2D, metabolic syndrome, hypertension, dyslipidemia, hyperinsulinemia, coronary artery disease, cardiovascular disease, osteoarthritis, chronic kidney disease, and numerous cancers. It is also linked to non-alcoholic steatohepatitis, sleep apnea, depression and other psychiatric disorders. Studies have also shown that obesity may have an impact on cognitive function and that a higher BMI may increase the chance of dementia or other cognitive impairments in later life (43).

Obesity and being overweight are known diabetes and prediabetes risk factors. However, these disorders can affect people with lower BMIs as well. The fasting triglyceride and glucose parameters have been proposed as alternative surrogate markers for identifying insulin resistance and diabetes (22, 27–30). Our study supports this finding, and all TyG-related parameters predict individuals with prediabetes (AUC > 0.5). On a large scale, when the AUC is equal to 1, it indicates faultless predictive power, and an AUC ≤ 0.55 means that the predictive power of a parameter is not better than chance (44). TyG-BMI showed the highest OR for prediabetes occurrence in normal-weight men. However, as determined by the AUC, its predictive value was not the highest compared to the other indices. The TyG-WHTR had the highest AUC value in men and women and was selected as the primary index for predicting prediabetes. The cut-off points indicated sensitivity and specificity values between 66 and 76% for both sexes, thus reducing false-positive and false-negative cases. TyG-WHTR proved significantly different from all obesity-related parameters in men and was significantly higher than the remaining TyG-related indices. It also had the highest AUC and was significantly different from all other indices in the obese population (Supplementary Tables S1, S2). Therefore, TyG-WHTR could potentially be adopted for identifying prediabetes in normal-weight and Ow/Ob men. Many TyG-related parameters were assessed for the predictive value of prediabetes and diabetes. However, no evidence has been published regarding the relationship between TyG-WHTR and prediabetes in normal-weight individuals. However, in recently published studies, TyG-WHTR exceeded the commonly used anthropometric markers in predicting diabetes (45, 46).

The TyG-WHTR index’s capacity to detect prediabetes was contrasted with FPG’s to determine whether it could be a useful screening tool. The results demonstrated that the predictive abilities were all significantly improved. These results indicate that the TyG-WHTR index can act similarly to FPG. Hence, TyG-WHTR can, alongside FPG, function in screening individuals for prediabetes. Knowing that advanced age poses an additional risk for prediabetes (11), further age stratification of the participants demonstrated that TyG-WHTR proved higher predictive probability with advanced age in normal-weight men and obese men and women, highlighting the efficiency of TyG-WHTR in predicting prediabetes.

TyG-WC was previously reported as the best predictor of prediabetes or diabetes (47). TyG-BMI was also suggested as the best index for detecting prediabetes in adults of either sex (25). Further, TyG was suggested as a good index for predicting insulin resistance and prediabetes (29, 31). We have found the TyG-WHTR to be the most effective index for prediabetes prediction. These disparities could be attributed to the ethnic diversity of the populations studied. Nonetheless, the overall conclusion from our findings and previously published data is that triglyceride-glucose (TyG)-related parameters outperform obesity parameters alone. The clinical significance of TyG-WHTR rests in its ability to identify persons at risk of developing prediabetes before symptoms appear. By recognizing these patients early, healthcare providers can take steps to prevent or delay illness onset through lifestyle changes like diet and exercise, or medication interventions if necessary. Furthermore, TyG-WHTR can be used to assess the efficacy of programs targeted at lowering the risk of prediabetes in people of normal weight. A patient, for example, may begin an exercise and diet regimen in order to reduce their waist circumference and improve their glucose and lipid levels. Their TyG-WHTR score can be tracked over time to evaluate the intervention’s effectiveness and make any necessary modifications.

In some cases, the TyG-WHTR may be considered superior to fasting glucose or HbA1c because: (1) fasting glucose and HbA1c may only show abnormalities after significant metabolic damage has occurred, whereas TyG-WHTR can detect early metabolic changes when interventions are more likely to be effective. (2) TyG-WHTR ca be a better predictor of prediabetes than fasting glucose or HbA1c. This is due to the fact that TyG-WHTR considers both triglyceride and glucose levels, which are both independent risk factors for dysglycemia. (3) TyG-WHTR may be more responsive to metabolic state changes than fasting glucose or HbA1c. For example, if a patient improves their diet and exercise habits, their TyG-WHTR score may improve even if their fasting glucose or HbA1c levels stay unchanged. (4) TyG-WHTR is a straightforward computation that involves only basic laboratory tests and measures found in most healthcare facilities. HbA1c testing, on the other hand, might be more expensive, and not all healthcare settings have the necessary equipment or competence. One of the strengths of our study is the large sample size. Indeed, according to the Qatar Planning and Statistics Authority, the population of Qatar at the end of April 2022 was 2,773,598 people (accessed on 22nd of May, 2022),^2^ with Qataris accounting for approximately 12% of the total (i.e., 333,000 individuals). Moreover, in 2015, individuals under 19 made up 47% of all Qatari nationals,^3^ and if this percentage did not change in 2022, approximately 176,500 individuals would be adults and would be eligible for our study. As a result, our study (6,000/176,500 = 0.03) has statistically significant power. Additionally, the data we used was obtained from a well-phenotyped cohort from the general population. Furthermore, our study is the first to compare the ability of obesity indices and TyG-related parameters to identify prediabetes in normal-weight individuals in a Middle Eastern population. It is worth noting that T2D is a major public health burden in the Middle East, and early detection of prediabetes in obese and normal-weight individuals is thus critical for implementing strategies to prevent its progression to T2D.

Given the shared environmental factors and lifestyle habits, as well as genetic background and ethnicity among many Middle Eastern countries, particularly the Gulf Cooperation Council nations (Qatar, Bahrain, Saudi Arabia, United Arab Emirates, Kuwait, and Oman), our findings may perform similarly in many of these countries.

The main limitation of our study is the cross-sectional design, which does not allow the use of the findings to predict future prediabetes. However, the QBB has recently started to call back the participants for a 5-year follow-up, which will open new avenues for assessing the predictive ability of the different indices longitudinally. We also did not adjust for parameters such as smoking status, medication, or physical activity. Finally, the findings of the present study may not be generalizable to all populations due to the ethnic and geographic characteristics of the study population.

## Conclusion

Based on our results, factoring in waist-to-height ratio with simple biochemical measurements of triglyceride and glucose proved to be the best indicator of prediabetes in normal-weight, overweight and obese men, in addition to outperforming most obesity indices in women and having similar predictive effects to FPG in both normal-weight and overweight/obese men and women. We suggest that TyG-WHTR be used in clinical practice as part of routine check-ups as an asserting indicator to FPG for predicting prediabetes in men. Further studies are warranted to confirm the predictive value of these parameters across varying ethnicities.

## Data availability statement

The raw data supporting the conclusions of this article will be made available by the authors, without undue reservation. Clinical, anthropometric, demographic and genetic data can be obtained from the Qatar biobank according to the applied rules.

## Ethics statement

The institutional review board approved the current project at the Qatar Biomedical Research Institute (IRB number: 2017–001) and QBB (IRB number: Ex‐2018‐Res‐ACC‐0123‐0067). All participants gave written informed consent for their data and biospecimens to be used in medical research. The patients/participants provided their written informed consent to participate in this study.

## Author contributions

NA, EH, and AA performed the statistical analysis. NA and AA interpreted the results and wrote the manuscript. HB revised the statistical analysis. AA conceived and designed the study, was the guarantor of this work, had full access to all the data in the study, and took responsibility for the data’s integrity and the data’s accuracy. All authors reviewed the results, edited the manuscript, and approved the final version.

## Funding

This project was funded by intermural grants from Qatar the Biomedical Research Institute to AA and from the Qatar Computing Research Institute to HB.

## Conflict of interest

The authors declare that the research was conducted in the absence of any commercial or financial relationships that could be construed as a potential conflict of interest.

## Publisher’s note

All claims expressed in this article are solely those of the authors and do not necessarily represent those of their affiliated organizations, or those of the publisher, the editors and the reviewers. Any product that may be evaluated in this article, or claim that may be made by its manufacturer, is not guaranteed or endorsed by the publisher.

## Footnotes

^1^ http://www.stata.com

^2^ https://www.psa.gov.qa/en/Pages/default.aspx

^3^ https://gulfmigration.org/qatar-population-nationality-qatari-non-qatari-five-year-age-group-2015/
