# Supplementary material for: The triglyceride glucose-waist-to-height ratio outperforms obesity and other triglyceride-related parameters in detecting prediabetes in normal-weight Qatari adults: A cross-sectional study
Source: Front Public Health. 2023 Apr 6;11:1086771. doi: 10.3389/fpubh.2023.1086771 (PMC10117653; doi:10.3389/fpubh.2023.1086771)
Supplement: Supplementary file 1 [file Data_Sheet_1.docx]

**Supplementary Material**

**The Triglyceride Glucose-Waist-to-height ratio outperforms obesity and other triglyceride related parameters in detecting prediabetes in normal-weight Qatari adults: a cross-sectional study**

Neyla S. Al Akl ^1^, Elias N. Haoudi ^2^, Halima Bensmail ^3^, Abdelilah Arredouani ^1*^

| **Table S1:** **ROC curve analyses for each index in predicting prediabetes in Ow/Ob participants stratified by gender** | | | | | | | | | | | | |
| --- | --- | --- | --- | --- | --- | --- | --- | --- | --- | --- | --- | --- |
| **Gender** | **Men (2150)** | | | | | | **Women (2584)** | | | | | |
| **ROC** | AUC (95%CI) | pValue | Cut-off | Sens(%) | Spec(%) | Youden index | AUC (95%CI) | pValue | Cut-off | Sens(%) | Spec(%) | Youden index |
| **WC** | 0.65 (0.63-0.68) | <0.0001 | ≥97.5 | 63% | 62% | 0.253 | 0.72 (0.70-0.73) | <0.0001 | ≥87.5 | 72% | 62% | 0.337 |
| **WHTR** | 0.68 (0.65-0.70) | <0.0001 | ≥0.55 | 72% | 58% | 0.294 | 0.73 (0.72-0.75) | <0.0001 | ≥0.55 | 73% | 63% | 0.361 |
| **VAI** | 0.61 (0.59-0.63) | <0.0001 | ≥1.17 | 74% | 45% | 0.191 | 0.65 90.63-0.68) | <0.0001 | ≥1.26 | 65% | 60% | 0.243 |
| **LAP** | 0.66(0.64-0.69) | <0.0001 | ≥39.15 | 71% | 56% | 0.272 | 0.72 (0.70-0.74) | <0.0001 | ≥30.02 | 76% | 59% | 0.349 |
| **TyG** | 0.66(0.64-0.69) | <0.0001 | ≥8.4 | 73% | 53% | 0.263 | 0.71(0.69-0.73) | <0.0001 | ≥8.3 | 75% | 58% | 0.326 |
| **TyG-BMI** | 0.65(0.63-0.67) | <0.0001 | ≥261.72 | 61% | 65% | 0.254 | 0.70(0.68-0.72) | <0.0001 | ≥262.12 | 73% | 58% | 0.310 |
| **TyG-WC** | 0.70(0.68-0.72) | <0.0001 | ≥848.65 | 65% | 66% | 0.313 | 0.75(0.73-0.77) | <0.0001 | ≥730.61 | 77% | 62% | 0.398 |
| **TyG-WHTR** | 0.71(0.69-0.74) | <0.0001 | ≥4.94 | 67% | 67% | 0.338 | 0.77(0.75-0.78) | <0.0001 | ≥4.73 | 74% | 68% | 0.418 |
| Overweight/Obese: Ow/Ob, ROC: Receiver Operating Curve; AUC: Area under the curve; WC: Waist Circumference, WHTR: Waist Height-Ratio, VAI: Visceral Adiposity Index, LAP: Lipid Accumulation Product, TyG: Triglyceride Glucose, TyG-BMI: TyG related to BMI, TyG-WC :TyG related to WC, TyG-WHTR: TyG related to WHTR | | | | | | | | | | | | |

| **Table S2: Pairwise comparison of AUC to TyG-WHTR in obese Participants** | | | | | | |
| --- | --- | --- | --- | --- | --- | --- |
|  | **Men (2150)** | | | **Women (2584)** | | |
|  | **Differences between AUC** | **95% CI** | **P value** | **Differences between AUC** | **95% CI** | **P value** |
| **WC** | 0.07 | (0.06-0.08) | <0.0001 | 0.06 | (0.05-0.07) | <0.0001 |
| **WHTR** | 0.03 | (0.02-0.04) | <0.0001 | 0.03 | (0.02-0.004) | <0.0001 |
| **VAI** | 0.10 | (0.08-0.12) | <0.0001 | 0.11 | (0.09-0.13) | <0.0001 |
| **LAP** | 0.05 | (0.0.4-0.06) | <0.0001 | 0.04 | (0.03-0.05) | <0.0001 |
| **TyG** | 0.04 | (0.02-0.07) | <0.0001 | 0.06 | (0.04-0.08) | <0.0001 |
| **TyG-BMI** | 0.06 | (0.05-0.07) | <0.0001 | 0.06 | (0.05-0.08) | <0.0001 |
| **TyG-WC** | 0.01 | (0.008-0.021) | <0.0001 | 0.01 | (0.008-0.019) | <0.0001 |
| Overweight/Obese: Ow/Ob, AUC: Area Under the Curve; WC: Waist Circumference, WHTR: Waist Height-Ratio, VAI: Visceral Adiposity Index, LAP: Lipid Accumulation Product, TyG: Triglyceride Glucose, TyG-BMI: TyG related to BMI, TyG-WC :TyG related to WC, TyG-WHTR: TyG related to WHTR | | | | | | |
